# A Paper-Based Ion-Selective Organic Electrochemical Transistor for Highly Sensitive Determination of Creatinine and Potassium

**DOI:** 10.1021/acsomega.5c04973

**Published:** 2025-08-05

**Authors:** Ariadna Dasca Beneito, Andrés Alberto Andreo Acosta, Andrés F. Sierra, Pascal Blondeau, Pablo Ballester, Jordi Riu, Francisco Javier Andrade

**Affiliations:** † Dept. Química Analítica i Química Orgànica, 16777 Universitat Rovira i Virgili (URV), Carrer de Marcel·lí Domingo, 1, 43007 Tarragona, Spain; ‡ 202569 Institute of Chemical Research of Catalonia (ICIQ), Av. Països Catalans, 16, 43007 Tarragona, Spain; § 117370 Catalan Institution for Research and Advanced Studies (ICREA), Pg. Lluís Companys, 23, 08010 Barcelona, Spain

## Abstract

A novel paper-based ion-selective organic electrochemical transistor (IS-OECT) for the detection of potassium and creatinine is presented. First, ion-selective membranes are cast onto thick-film transistor channels to create highly sensitive and selective ion sensors. Since optimum performance is obtained at 0 V gate voltage, several sensors can be used in parallel with a single gate connected to a common grounded source electrode. Then, to further demonstrate the detection capabilities, the IS-OECTs were integrated with a purposely designed differential amplifier. This allows the conversion of current signals into a voltage output, facilitating comparison with potentiometric systems and the use of low-cost commercial data acquisition platforms. The evaluation of the performance in artificial serum is performed in clinically relevant ranges, which comprise 2.4–5.75 mM for potassium and 30–140 μM for creatinine. These results highlight the potential of the IS-OECT framework as a cost-effective, portable, and reliable solution for point-of-care diagnostics.

## Graphical abstract

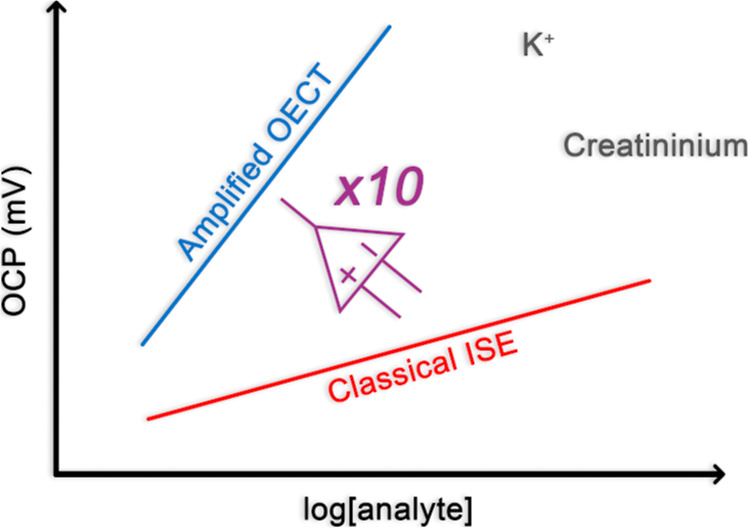

## 1 Introduction

The levels of potassium and creatinine in serum are an essential part of the routine for monitoring several health parameters, such as kidney and cardiovascular function, and for the diagnostic and management of chronic kidney disease (CKD). The accurate determination of potassium is critical. Normal serum levels range from 3.5 to 5.5 mM, and both hypokalemia (<3.5 mM) and hyperkalemia (>5.5 mM) are life-threatening conditions.^1^ Creatinine levels, used to estimate the glomerular filtration rate, have normal values ranging from 45 to 110 μM. Levels above 140 μM are indicators of acute renal dysfunction. When kidney function deteriorates, creatinine levels must be monitored to track the progress of CKD,^2,3^ while K^+^ levels must be closely checked to avoid severe health problems. CKD is an often asymptomatic silent threat, normally appearing as a comorbidity of diabetes and hypertension, and is projected to be the fifth leading cause of death by 2040.^4^ For this reason, with the growing role of remote monitoring platforms in digital health, there is an increasing demand for accurate, simple, and affordable tools to measure K^+^ and creatinine levels in the point of need.

The quantification of creatinine is most often performed either with the colorimetric Jaffé reaction^5^ or with enzymatic methods.^6^ None of these approaches can be easily implemented outside the lab. Potassium, on the other hand, is typically measured using potentiometric ion-selective electrodes (ISEs). Potentiometry is a very attractive tool for the point of need because the sensors are simple, robust, and compact and can be built on low-cost substrates.^7^ For that reason, there has been many efforts devoted to the development of mass-producible^8^ and disposable wearable potentiometric sensors.^9^ Our group has reported sensors for decentralized monitoring of lithium,^10^ potassium,^1^ and glucose^11^ and, more recently, a novel sensor for the determination of creatinine.^12^

The benefits of these platforms have been demonstrated in the lab. However, their implementation in real settings still has significant challenges. From an instrumental point of view, a major drawback of potentiometry is the detection of very small and weak signals. Due to the limitations of Nernstian sensitivity, relevant clinical variations are reduced to very small changes in signal, often requiring the precise detection of sub-mV values.^13^ Furthermore, since measurements must be performed with high input impedance instruments, the signal has a very low power, which makes it more vulnerable to capacitive losses, drift, and environmental noise.^14^ Therefore, simple, rugged, and highly affordable electronic platforms that are nowadays widely available on the market cannot be easily adopted for this use. Common analog readers, for example, have a 12-bit analog-to-digital (ADC) converter. This can discriminate voltages down to a few millivolts, which is not enough for many clinical applications. If the sensor can generate larger signals with higher power, the use of these simpler, cheaper, and more rugged instrumentation would facilitate the adoption of tools for the point of need.

Organic electrochemical transistors (OECTs) have emerged as an interesting way to overcome some of these issues. OECTs have drawn increasing attention during the past decade because of their high sensitivity, simplicity, and versatility.^15,16^ Two key advantages of OECTs are their unique detection mechanism, which combines signal transduction and amplification, and a simple instrumental setup.^17^ Like any other transistor, the OECTs are made with three electrodes. Two of them, the source (S) and the drain (D), are connected through an organic conducting material, typically poly­(3,4-ethylenedioxythiophene)-poly­(styrenesulfonate) (PEDOT:PSS), which forms the channel.^18^ While PEDOT alone is normally in a reduced, nonconductive form, in the presence of PSS, it becomes a good electronic conductor.^19^ The electrostatic effect produced by the negative sulfonate groups of PSS stabilizes the formation of positive charge carriers (holes) in PEDOT, i.e., the sulfonate groups shift the equilibrium toward the oxidized, conductive form (PEDOT^+^). For this reason, the incorporation of cations into the channel shields the effect of the sulfonate groups,^20^ shifting the equilibrium toward the reduced form of PEDOT. This cation-induced reduction in the concentration of holes, which produces a marked decrease in the electrical conductivity of the channel, is the working principle of OECTs.^21^ In practice, the channel and a third electrode—the gate (G)—are immersed in an electrolyte solution. A voltage applied to the gate (*V*_g_) is used to control the migration of cations into and out of the channel, which results in the electrical conductivity of the channel being controlled by *V*_g_.^22^ Therefore, when a fixed voltage is applied between S and D (*V*_d_), the magnitude of the current in the channel (*I*_d_) will depend on *V*_g_.^23^ The transconductance (*g*_m_) measures the change in *I*_d_ produced by changes in *V*_g_. Because of their unique characteristics, OECTs show *g*_m_ values significantly higher than other types of transistors, i.e., small changes in *V*_g_ create large variations in *I*_d_. Since working currents normally range from hundreds of μA to several mA, low power voltage signals are converted into a current response with a significantly higher power.^23^ In a recent work, for example, we have shown that suitable optimization of the ion-selective membrane in combination with thick film channel technology^24,25^ allows the development of ion-selective OECTs with a sensitivity of up to 2.5 mA/dec. The power output of the OECT signal, on the order of 1 mW, is many orders of magnitude higher than potentiometry. This type of signal is less vulnerable to the multiple sources of noise that are commonly encountered, for example, when sensors must work onto the skin of people.^26^

$$g_{m}=\frac{\partial I_{d}}{\partial V_{g}}$$

A practical limitation of OECTs is that the output signal is a current, which is more difficult to read with conventional instrumentation. Commercial electronic platforms have multiple analog inputs to read voltages. Therefore, converting the ion-selective OECT signals into a voltage output can help to simplify the instrumentation.^27^ Additionally, this conversion can help compare the results to potentiometric techniques. Many works that transform OECT signals into potentials have been reported. For instance, Shiwaku et al.^28^ used organic thin film transistors coupled with an amplifier circuit to increase the sensitivity of potassium to 160 mV/dec.

In this work, we propose an alternative approach using a very simple circuit fitted with a low-cost, high-gain, and low-noise instrumental amplifier to transform the current output of an OECT into a voltage. We applied this circuit to an OECT ion-sensing setup for the determination of potassium and creatininium in clinical ranges. The results show that the sensitivity of the OECT systems can reach values of 540 mV/dec, which is almost 10 times the Nernstian sensitivity. Furthermore, since the current-to-voltage circuit produces a low-impedance, higher power signal, a simple, low-cost device such as an Arduino Nano platform can be easily used to read the signal. This demonstrates the potential of this approach for developing portable and wearable devices on a large scale.

## 2 Experimental Section

Details of the chemicals used, preparation of solutions and ion-selective membrane (ISM) cocktails, and fabrication of ion-selective electrodes (ISE) can be found in the Supporting Information.

### 2.1 Fabrication of OECTs

The construction of thick-film OECTs has been reported elsewhere, and the basic details can be seen in Figure 1A.^24,29^ Briefly, to make the source (S) and drain (D) electrodes, a 0.5 mm wide adhesive tape is placed along a photography-quality paper (200 μm thick). This ensemble is then coated by sputtering with a 100 nm thick layer of Au. After peeling off the adhesive tape, two Au electrodes separated by a 0.5 mm gap remain in the paper. This paper is cut into 2.5 cm long pieces, and a hydrophobic adhesive mask with a 3 mm diameter window is added on top, leaving exposed part of the electrodes and the 0.5 mm gap between them. The conductive channel is created by drop casting 1.5 μL of a filtered PEDOT:PSS solution to completely cover this window. This system is first dried at room temperature and then in an oven at 100 °C for 20 min. Thereafter, 1 μL of DMSO is added onto the PEDOT:PSS channel and left to dry again to improve the conductivity.^15^ Finally, the sensor is rinsed with distilled water and dried at room temperature. Before casting the membrane, the PEDOT:PSS channel is conditioned by immersion in a 10^–2^ M solution of the cation of interest (creatininium ion or K^+^) for 2 h. Lastly, this system is rinsed with distilled water and dried for 3 h at room temperature. Once the channel is dry, a 5 μL aliquot of the optimized ion-selective membrane (ISM) cocktail is drop cast onto the channel and dried for 5 min. The procedure is repeated until a total of 15 μL of the ISM has been added, and the system is dried overnight at room temperature. Prior to its use, the functionalized channel is conditioned by immersion in a 10^–1^ M solution of the ion of interest. Thereafter, the system is rinsed with distilled water and is ready to use (for further experimental details, see the Supporting Information).

**Figure 1:**
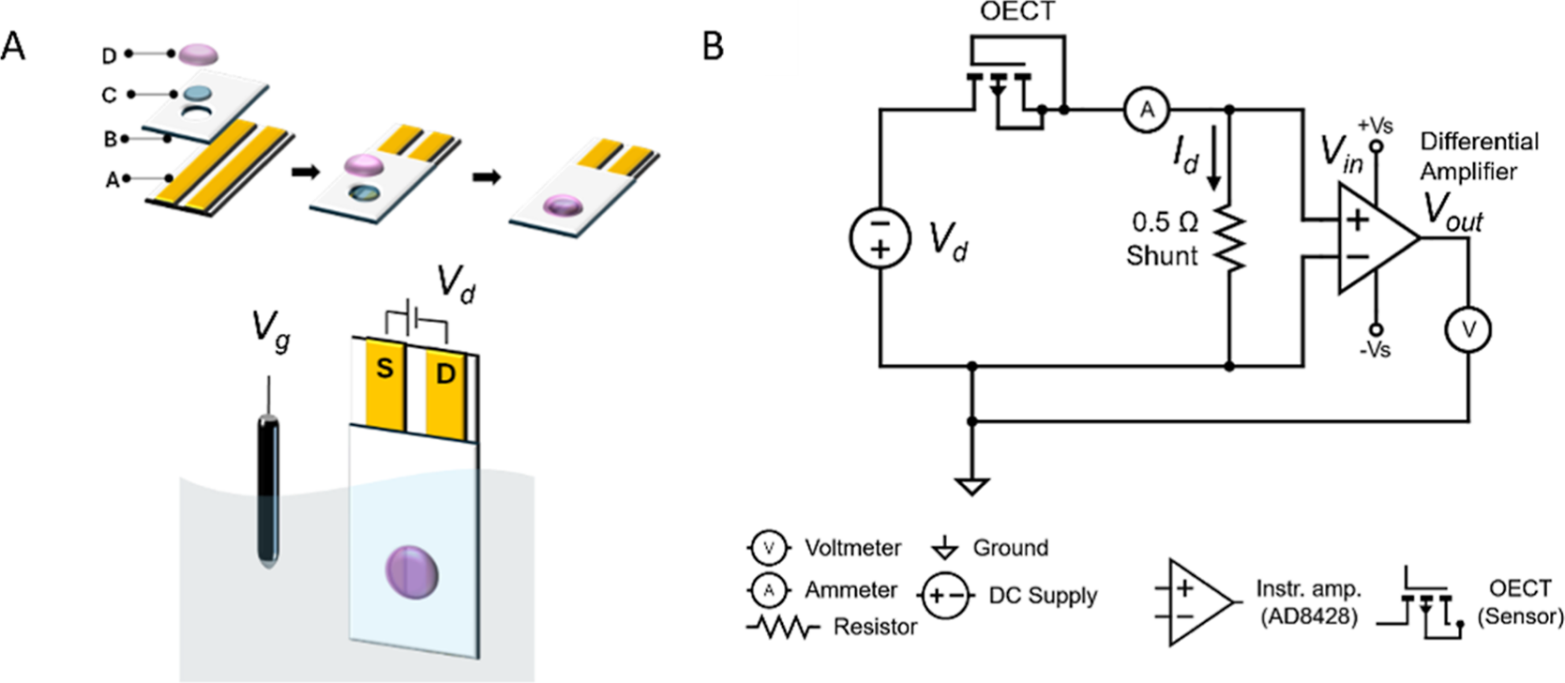
(A) Schematics of the construction of the channel (A: Drain and source electrodes, B: Mask, C: PEDOT:PSS, and D: Ion-selective membrane) with the electrical circuit (S: Source, D: Drain, *V*_*g*_: Gate voltage, and *V*_*d*_: Drain voltage). (B) Circuit diagram for the current-to-voltage amplifier with decoupled DC power supply (*V*_in_: Input voltage).

A 2 mm-diameter Ag/AgCl flat tip probe (Warner instruments, 641311) was used as the gate electrode in all of the experiments. All measurements were performed in a 0.05 M acetic acid/magnesium acetate buffer at pH 3.8 to shift the equilibrium of creatinine (p*K*_a_ = 4.8) to the formation of the creatininium cation.^12^

### 2.2 Voltage Transduction/Amplification

Current was converted into a voltage using a laboratory-assembled circuit that contains a shunt resistor (Figure 1B) connected to the inputs of a differential amplifier. In essence, the voltage drop produced by *I*_d_ passing through a 0.5 Ω shunt resistor was amplified through the dedicated amplifier. An AD8428 differential amplifier (Analog Devices, Inc., Wilmington, MA, USA) was used, since it provides a fixed 2000 voltage gain with a compact, low-noise output.^30^ A functional block diagram and schematics can be seen in Figures S2 and S3. The amplifier was powered with ±18 V, and 0.1 μF decoupling capacitors were used to reduce noise. Further details of the instrumentation can be found in Supporting Information.

## 3 Results and Discussion

The current-to-voltage transduction-amplifying circuit is shown in Figure 1B. The channel current (*I*_d_) produces a voltage drop in the shunt resistor that is amplified by a differential amplifier connected to both ends of the resistor. This circuit must be designed to maximize the voltage drop in the shunt resistor (*V*_In_), without interfering with the transistor operation (i.e., *V*_In_ must be negligible compared to *V*_d_). Under typical operating conditions, our IS-OECT runs with *I*_d_ = 10 mA and *V*_d_ = −0.4 V. Therefore, a 0.5 Ω shunt resistor was used, since it would produce a maximum *V*_In_ of 5 mV, a value that can be considered negligible with respect to *V*_d_. Both ends of the shunt resistor are connected to the inputs of an instrumentation amplifier (AD8428) with a high common-mode rejection ratio, designed to address low-level measurements with ultralow levels of noise. This system has a fixed 2000× gain, i.e., a voltage output (*V*_out_) of 2 V/mV. A calibration of the voltage output vs *I*_d_ using the circuit of Figure 1B shows—as expected—a response of 1 V/mA, with a noise level in the order of 0.76 mV, which corresponds with a measured level of noise in the current of 0.51 μA. It should be mentioned that further reduction of the noise could be achieved by using additional features, such as noise compensation inputs present in the amplifier. Nevertheless, this step was not explored in this work.

Optimization was first performed by using K^+^ OECTs. The time trace for increasing additions of K^+^ can be seen in Figure 2A. Due to the 1 V/mA amplification, the plots of *I*_d_ and *V*_out_ can be overlapped. For the current measurements, a slope of 0.496 mA/dec is observed, which is in line with the results previously reported. The corresponding amplified voltage response is 495.0 mV/dec. To compare this result with a traditional potentiometric sensor, the time trace of the open circuit potential (electromotive force, EMF) of a potassium ISE was compared with the voltage amplified signal of the OECT (Figure 2B). The EMF showed a better response time. However, as expected, the sensitivity is 52.0 mV/dec, which is almost ten times lower than that of the OECT. Figure 2C shows the overlapped calibration curves for K^+^ in the potentiometric and OECT-voltage mode. Similar results were obtained for the creatinine sensor, where the OECT-voltage mode yielded a response of 483.0 mV/dec, while the potentiometric mode showed a sensitivity of 55.3 mV/dec (Figure 2D).

**Figure 2:**
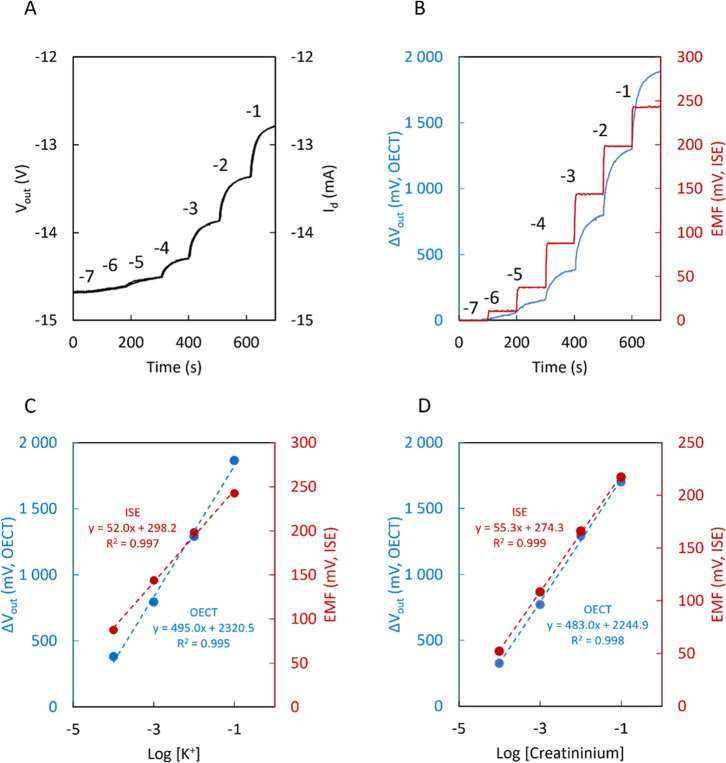
(A) Overlapped time traces for a K^+^ OECT. (B) Typical time trace of background subtracted *V*_out_ vs time for a K^+^ OECT and EMF vs time for a K^+^ ISE. (C) Corresponding calibration curve for a K^+^ OECT and a K^+^ ISE. (D) Comparison of the calibration curves for a creatininium OECT and a creatininium ISE. All the above experiments were performed at pH 3.8.

The uncertainty on the baseline—estimated as 3 times the standard deviation of the noise for a 60 s period—is 0.8 mV for the voltage-amplified OECT and 0.2 mV for the potentiometric sensor. Considering the differences in sensitivities, this suggests that the detectability using the voltage-OECT should be slightly better than the detection with the OCP system. Nevertheless, considering that the voltage amplifier circuit is an in-house, handmade circuit, these values are a preliminary proof of concept, showing that the amplification does not decrease the signal-to-noise ratio. Improvements to the electronics of the amplifying circuit could further enhance the signal-to-noise ratio obtained in this report. Further analytical characterization was performed to confirm that the amplification does not affect the performance: the sensor-to-sensor variability and the selectivity were assessed, and the results are consistent with previous reports (see Supporting Information).^29,31,32^

To highlight the potential clinical applications of IS-OECTs, calibration curves were performed in artificial serum, adjusted at pH = 3.8. Figure 3A displays the calibration curve for the K^+^ OECT in the range of 2.4–5.75 mM. For such a narrow concentration range, the data can be linearized, obtaining a sensitivity of 67.3 mV/mM. With this sensitivity, differences in concentration below 0.05 mM can be detected. Similarly, a calibration curve for creatininium was performed, in this case in the range of 30–140 μM (Figure 3B). Since the concentration range is much wider, linearization is not possible, and the logarithmic scale must be used. Creatininium shows a sensitivity of 376.8 mV/dec. It should be stressed that, in OECTs, changing the matrix composition may lead to changes in sensitivity. Noteworthily, this system also allows those variations in concentration below 0.05 mM.

**Figure 3:**
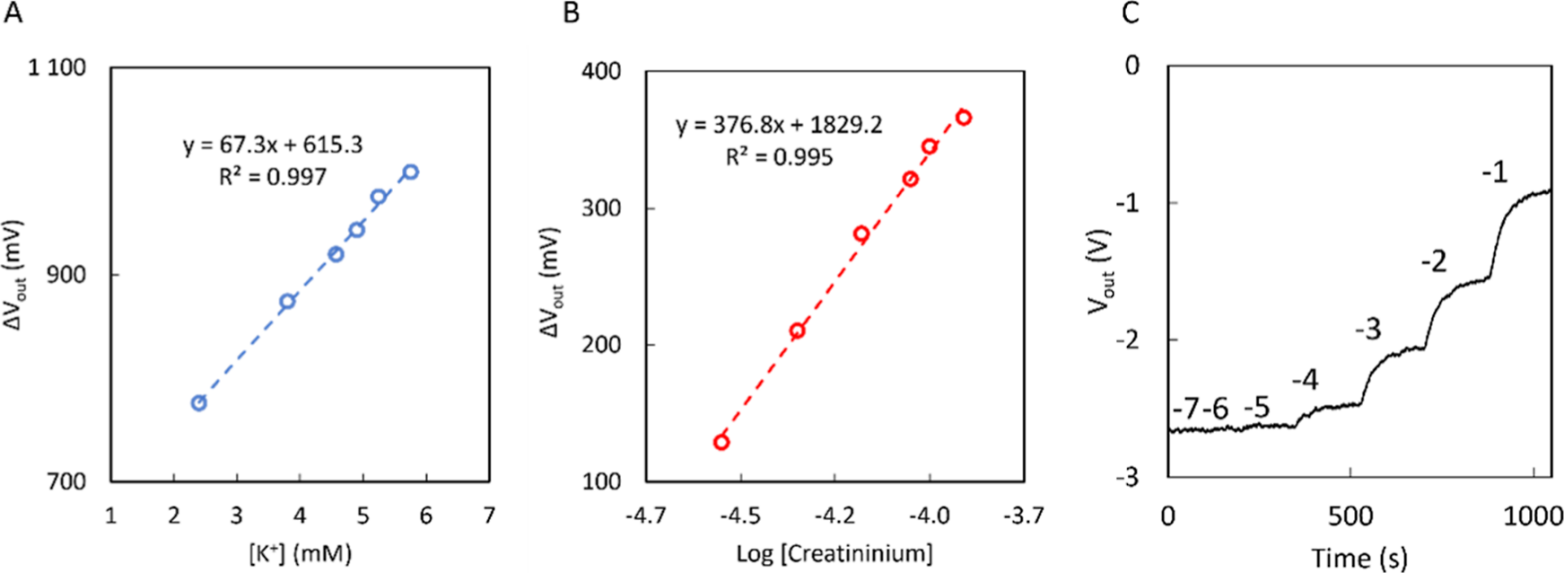
(A) Calibration curve linearized in the range of 2.4–5.75 mM for a potassium OECT. (B) Calibration curve from 30 to 140 μM for a creatininium OECT. (C) Typical time trace *V*_out_ vs time in the different additions in −2.5 V for a potassium OECT. All the above experiments were performed in artificial serum at pH 3.8.

To prove the potential of this setup in portable and wearable devices, a simple, low-cost Arduino Nano 33 BLE microcontroller fitted with a Bluetooth transmitter was used. The Arduino built-in analog-to-digital converter (ADC) was used to measure the amplified voltage signal. To secure the ADC input range, the reference voltage of the differential amplifier was offset to 12 V. The Arduino board was powered by a common lithium-polymer battery, and data were output via the Bluetooth Low Energy (BLE) protocol with basic functionality and retrieved with any general BLE analyzer smartphone app. A time trace for the K^+^ OECT is displayed in Figure 3C. Further analytical characterization was carried out regarding sensor-to-sensor variability with the calibration of three K^+^ OECTs (Figure S9). The slopes obtained for each sensor were comparable, with an average value of 515.8 mV/dec for the K^+^ OECT. The low variability, on the order of 5% in terms of sensitivity, is consistent with the previous results.

## 4 Conclusions

This paper reports a new ion-selective organic electrochemical transistor setup coupled to a signal-amplifying electronic circuitry. In particular, the use of an instrumentation amplifier such as AD8428 enhances the sensitivity of IS-OECTs up to 9 times compared to traditional ion-selective electrodes. The developed IS-OECT platform was successfully applied to the sensitive and selective detection of clinically relevant biomarkers such as potassium and creatininium. Detection of variations as low as 17 μM underscores its potential for point-of-care applications. The compact and portable nature of the device was further demonstrated via wireless measurements with an Arduino BLE board and a smartphone. These results provide the basis for highly sensitive platforms with point-of-care applications.

## Supplementary Material



## Notes

Additional experimental and sensor details, materials, and methods, including supplementary experimental assessments; preparation of reagents and solutions; sensor fabrication and conditioning protocols; instrumentation and experimental parameters; and further sensor characterization including variability and analytical performance evaluations (static resistance, noise, response time, intermediate precision, repeatability, and selectivity) (PDF)
