# Supplementary material for: A Paper-Based Ion-Selective Organic Electrochemical Transistor for Highly Sensitive Determination of Creatinine and Potassium
Source: ACS Omega. 2025 Aug 5;10(32):36475–80. doi: 10.1021/acsomega.5c04973 (PMC12368651; doi:10.1021/acsomega.5c04973)
Supplement: Supplementary file 1 [file ao5c04973_si_001.pdf]

Supporting information

A paper-based ion-selective organic electrochemical transistor for highly sensitive determination of creatinine and potassium

Ariadna Dasca Beneito,[1#] Andrés Alberto Andreo Acosta,[1#] Andrés F. Sierra[2] Pascal Blondeau,[1] Pablo Ballester,[2,3] Jordi Riu[1] and Francisco J. Andrade[1*]

[#] These authors equally contributed to this work.
[1] Universitat Rovira i Virgili (URV), Dept. Química Analítica i Química Orgànica. Carrer de Marcel·lí Domingo, 1, 43007 Tarragona, Spain
e-mail: franciscojavier.andrade@urv.cat
[2] Institute of Chemical Research of Catalonia (ICIQ). Av. Països Catalans, 16, 43007 Tarragona, Spain.
[3] Catalan Institution for Research and Advanced Studies (ICREA). Pg. Lluís Companys, 23, 08010 Barcelona, Spain.

Keywords: organic electrochemical transistor; transimpedance amplification; paper-based platform

Reagents and solutions

All chemicals used were purchased from Sigma – Aldrich (Merck, Spain). Channel construction: conductive inkjet ink poly(3,4-ethylenedioxythiophene)-poly(styrenesulfonate) 0.8% in H_2O. Dimethyl sulfoxide (DMSO) (H_2O ≤0.01%), ≥99.5% was used to deposit on top of the channel. Ion-selective membrane: potassium ionophore I ≥95.0%, potassium tetrakis[3,5-bis(trifluoromethyl)phenyl]borate ≥95.0%, poly(vinyl chloride) high molecular weight (PVC), 2-nitrophenyl octyl ether (NPOE) and anhydrous tetrahydrofuran ≥99.9% (THF). Tetra-aryl-substituted, monophosphonate-bridged calix [4]pyrrole for creatininium OECT was provided by the Ballester research group.[1]

Analytical grade salts for the standard solutions of NaCl, KCl and creatinine were used as well as buffer solution of 50 mM acetic acid/magnesium acetate tetrahydrate pH 3.8 for the background electrolyte solution.[2] The dilution of the PEDOT:PSS and the remain solution were prepared using double deionized water (Millipore Corporation, Bedford, MA). Artificial serum preparation (pH =7.4): NaCl (6.487 g/L), $NaHCO_3$ (2.436 g/L), $MgCl_2 \cdot 6H_2O$ (0.162 g/L), urea (0.150 g/L) and glucose (0.847 g/L).

Preparation of the ion-selective membrane cocktail

The optimal composition of the ISM for potassium was determined based on prior studies[3]. For K^+ OECT sensors, the ISM formulation included potassium tetrakis[3,5-bis(trifluoromethyl)phenyl]borate (0.5 wt.%) as the ion exchanger and potassium ionophore I (2 wt.%) as the ionophore. The polymeric matrix was composed of poly(vinyl chloride) (10.9 wt.%) and 2-nitrophenyl octyl ether (21.6 wt.%) as the plasticizer. The components were dissolved in tetrahydrofuran (1 mL/35 mg). For K^+ ISE and creatininium OECT sensors, the ISM composition

was optimized to enhance signal performance[2]. The formulation contained potassium tetrakis[3,5-bis(trifluoromethyl)phenyl]borate (1 wt.%) as the ion exchanger. For the ionophores, tetra-aryl-substituted monophosphonate-bridged calix[4]pyrrole was used in creatininium OECT sensors, while potassium ionophore I (3.2 wt.%) was used in K^+ ISE sensors. The polymeric matrix was comprised of poly(vinyl chloride) (31.9 wt.%) and 2-nitrophenyl octyl ether (63.9 wt.%) as the plasticizer. All components were dissolved in tetrahydrofuran (1 mL/35 mg). The ISM cocktails were sonicated for 10 minutes to ensure homogeneity and stored at 8°C.

Fabrication of conventional ion-selective electrodes (ISEs)

Carbon working electrodes for ISEs were fabricated following previous works by the group.[4] In short, a filter paper was coated with a single layer of carbon ink and dry in the oven for 20 minutes at 100°C. Once dried, paper was cut (8 mm x 20 mm) and covered with a water-resistant adhesive mask on the front and back (15 mm x 15 mm). The front mask has a circular window (radius = 1.5 mm), leaving part of the carbon exposed. To create the ISE, functionalization was carried out by casting ISM cocktail onto the window, part of which will be exposed directly to the sample. A 5 µL aliquot of the ISM cocktail was drop cast and let dry for 5 min. This process was repeated until a total of 15 µL of the cocktail had been added. The functionalized window was let dry overnight. Before measuring the electrode was conditioned by immersion in an aqueous solution of 10−1 M of the analyte under constant stirring for 1 h and was then rinsed with distilled water.

IS-OECT conditioning

To create the OECT channel, 1.5 µL of a filtered PEDOT:PSS solution was drop cast onto the window, covering all the exposed area. This was then dried in an oven (100°C, 20 min). To enhance the channel conductivity, 1 µL of DMSO was added to the window, then dried again (100°C, 15 min) and then rinsed with distilled water. Before functionalization, the channel was conditioned in an aqueous solution of 10−2 M KCl or in a buffer solution (pH 3.8) in a 10-2 M creatininium under constant stirring for 2 h. Finally, the channel was rinsed and left to dry for 3 h at room temperature. Functionalization was carried out by casting ISM cocktail onto the channel. A 5 µL aliquot of the ISM cocktail was drop cast and let dry for 5 min. This process was repeated until a total of 15 µL of the cocktail had been added. The functionalized channel was let dry overnight and before measurements, it was conditioned by immersion in an aqueous solution of 10−1 M KCl or in a buffer solution (pH 3.8) in a 10-1 M creatininium under constant stirring for 1 h and was then rinsed with distilled water.

Equipment

Sputtering system: The instrument is a magnetron sputtering system ATC Orion from AJA International Inc, Massachusetts – USA. For depositing 100 nm gold in two steps process: strike (Pressure = 30 mTorr, Gas flow rate = 20 sccm (Argon), Power = 50 W, Time = 10 s), and

deposition (Pressure = 3 mTorr, Gas flow rate = 20 sccm (Argon), Power = 200 W, Coat time = 384 s).

Keysight E3631A (Keysight Technologies, Santa Rosa, CA, USA) and TENMA 72-10505 (Element14®, Newark, NJ, USA) adjustable power supplies were used for the application of the gate and drain voltages. Keithley 6514 and Keithley 2100 (Keithley Instruments, Cleveland, OH, USA) high-performance electrometers were used for measure the output current/voltage. For all experiments, a 2x4 mm Ag/AgCl flat tip probe was used as the gate electrode (input voltage) (Warner INSTRUMENTS, Holliston, MA, USA).

Electrochemical measurements

All the experiments have been performed using a 50 mM acetic acid/magnesium acetate (pH 3.8) as a background electrolyte. The sensing framework is built using two different functionalized channels (one for each analyte) and a common gate. For the electrical connections, the source electrode of each transistor, together with the gate, are connected to a common ground since maximum transconductance was proven for $V_g = 0$ V.[5] The drain electrode (D) of each transistor is connected to an independent PSU.

Static resistance

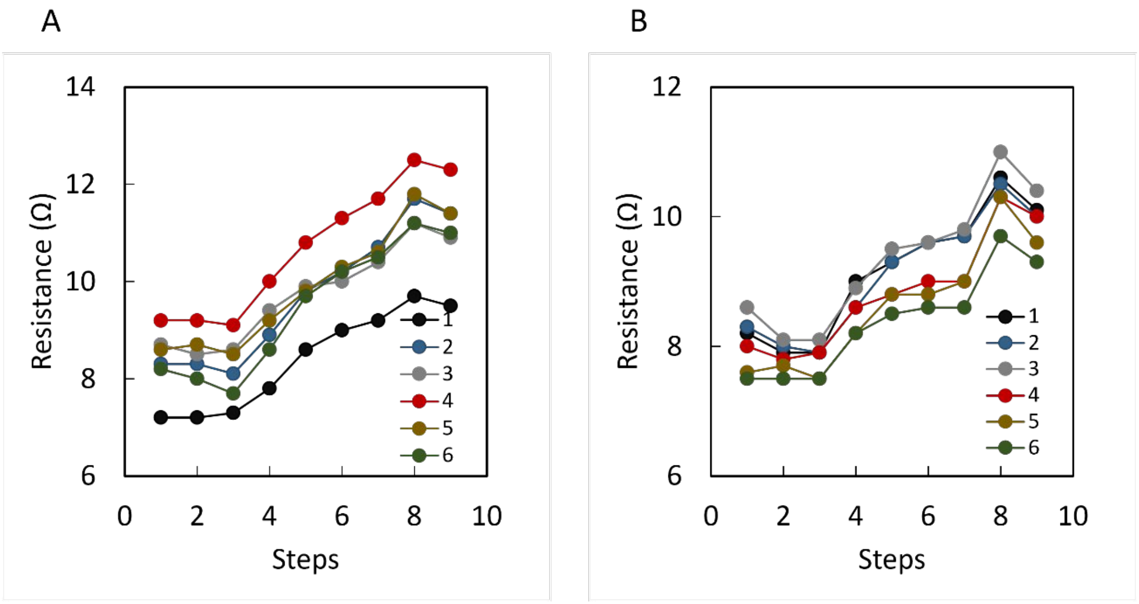

Figure S1. (A) Resistance characterization for each step of sensor creation (A) Potassium OECTs (B) Creatininium OECTs.

Instrumentation amplifier

Prior the measurement, the sensor signal was amplified by an AD8428 ultralow noise instrumentation amplifier (Analog Devices, Inc., Wilmington, MA, USA) with a set gain of 2000. Functional block diagram and schematics are shown below in Figures S2 and S3.

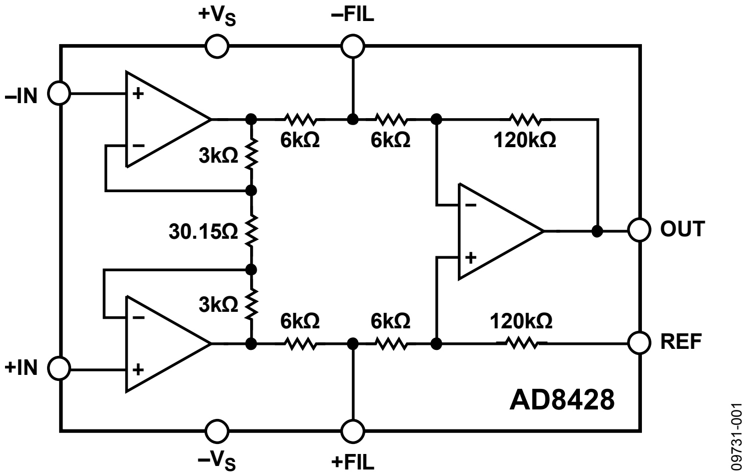

Figure S2. Functional block diagram of the AD8428 by Analog Devices, Inc. highlighting its internal components. Extracted from the technical datasheet.[6]

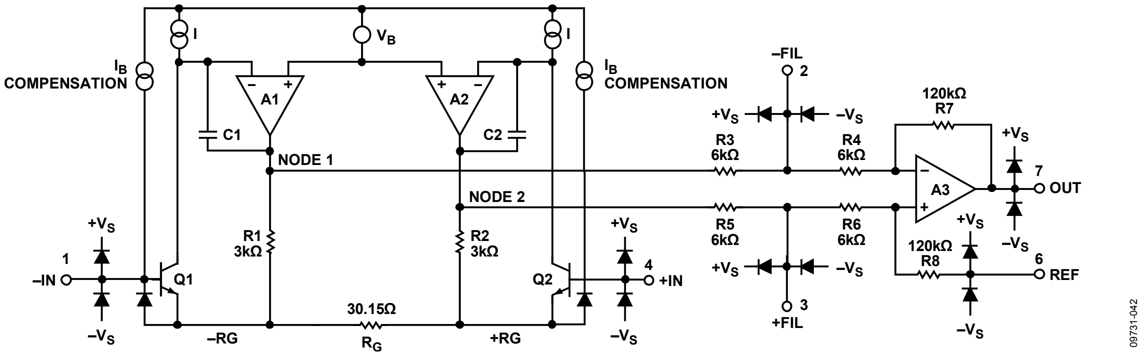

Figure S3. Simplified schematics of the AD8428 by Analog Devices, Inc. highlighting its internal components and features. Extracted from the technical datasheet.[6]

Noise

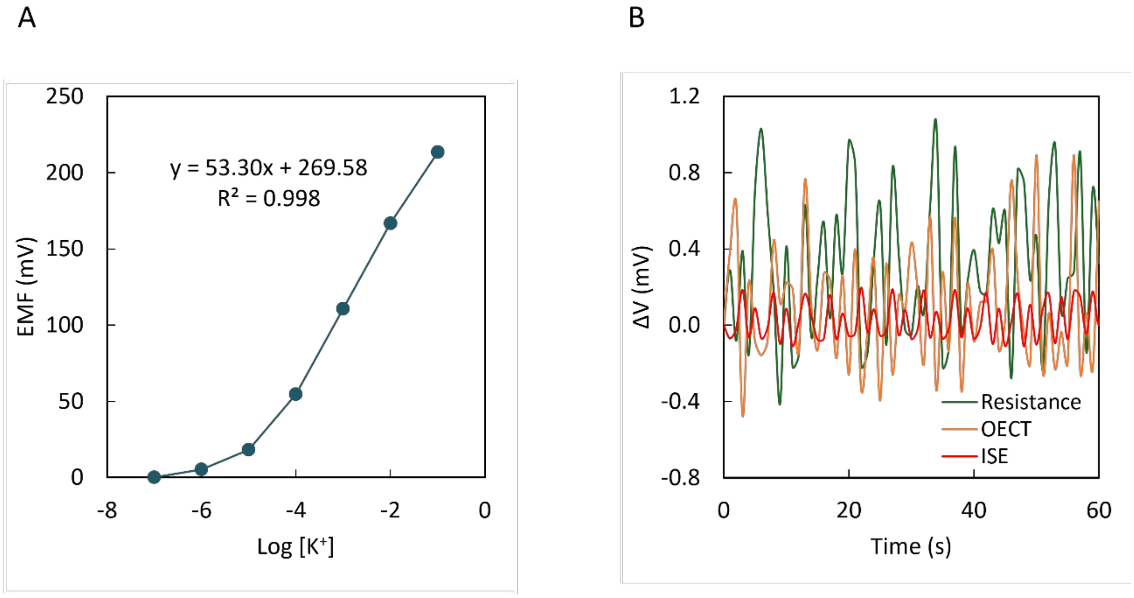

Figure S4. (A) Typical calibration for a potassium ISE, the linear range was calculated from 10^{-4}-10^{-1} M. (B) Baseline noise evaluation over time for a 15 Ω resistor (blue), an OECT with channel resistance of 12 Ω (orange) and an ISE (red). The results are shown as three times the standard deviation (3*σ) for 20 seconds: 1.5, 0.8 and 0.2 mV for a resistor, an OECT and an ISE, respectively.

Response time

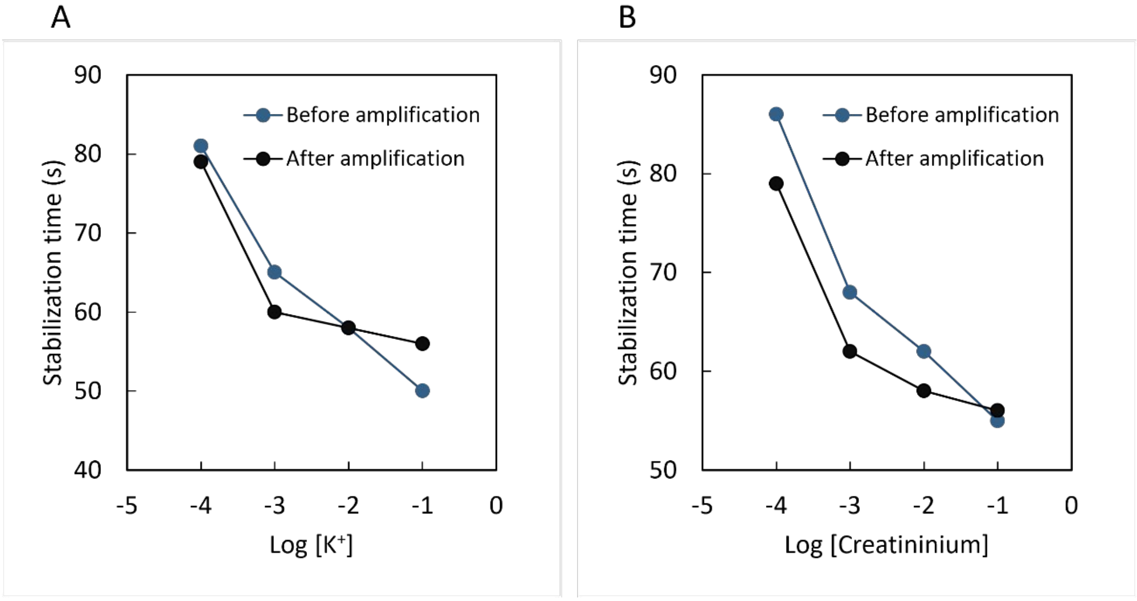

Figure S5. (A) Stabilization time *vs* concentration of each addition for each IS-OECT before and after amplification. (A) In a potassium OECT. (B) In a creatininium OECT.

Intermediate precision

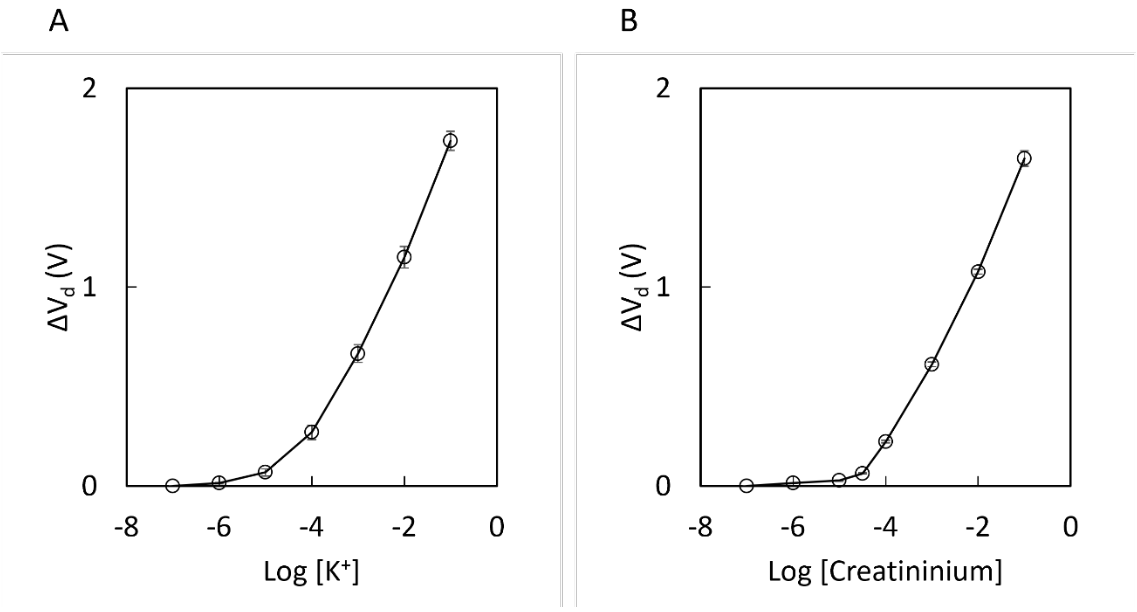

Figure S6. (A) Evaluation of intermediate precision of the IS-OECTs for 2 different sensors (A) In a potassium OECT. (B) In a creatininium OECT. The error bars correspond to standard deviation for 6 different sensors.

Repeatability

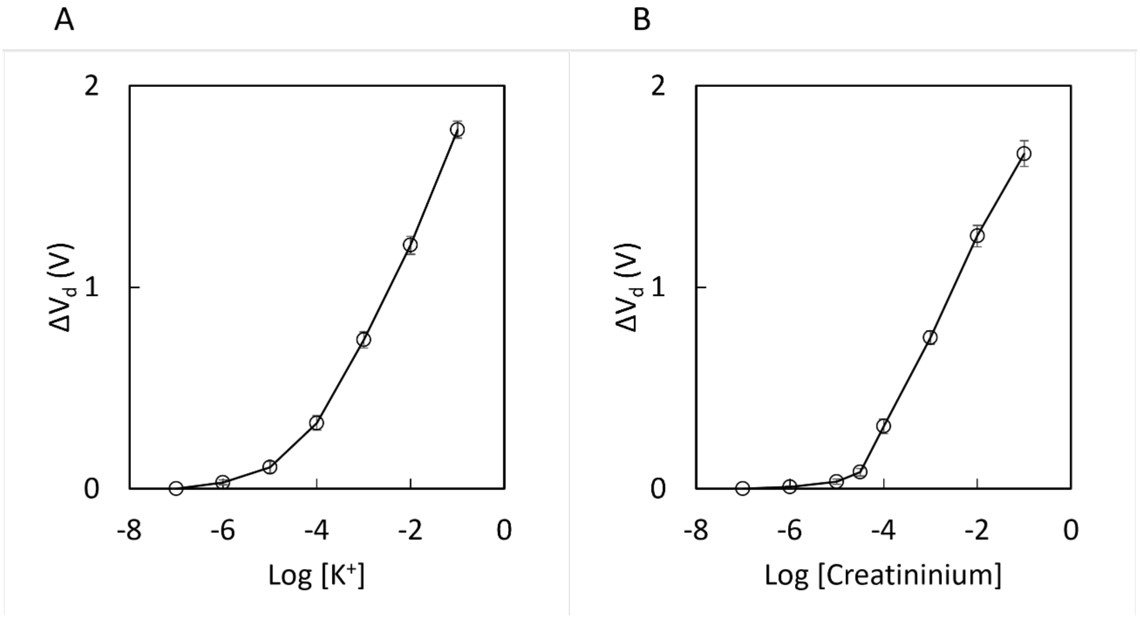

Figure S7. (A) Evaluation of repeatability of the IS-OECTs for 2 different sensors (A) In a potassium OECT. (B) In a creatininium OECT. The error bars correspond to standard deviation for seven consecutive calibrations.

Selectivity

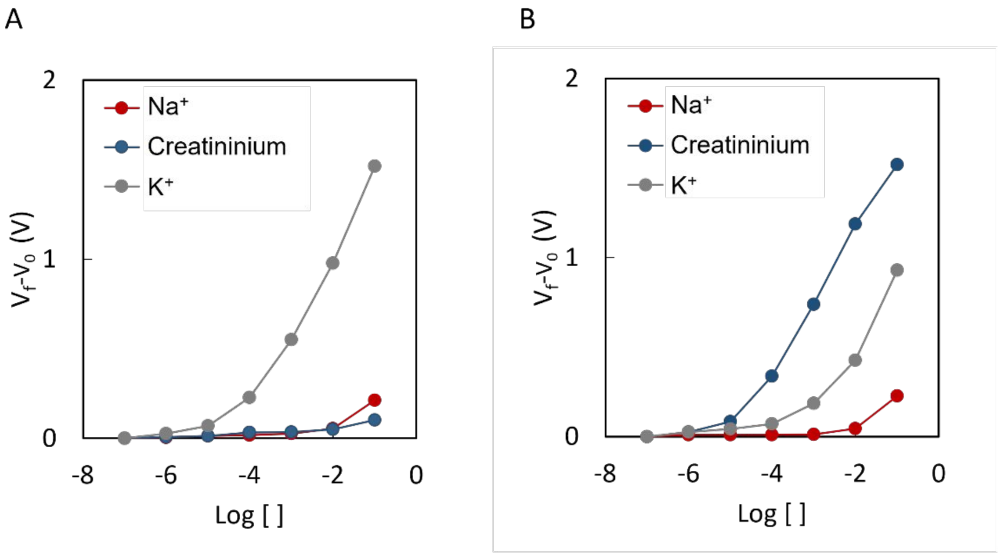

Figure S8. Selectivity studies: total response V_f-V_0 for the evaluation of interfering effect in the IS-OECT (A) In a potassium sensor. (B) In a creatininium sensor.

Measurements performed with Arduino board

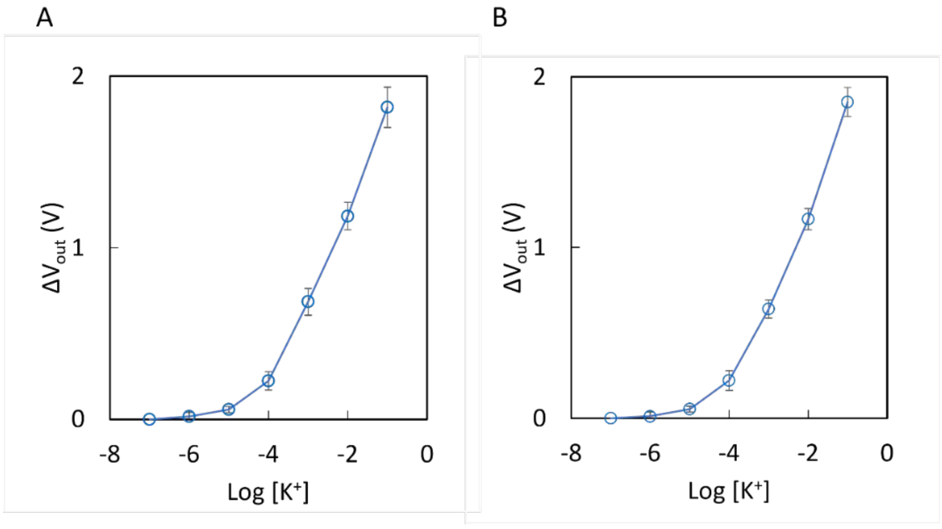

Figure S9. (A) Evaluation the intermediate precision for 3 different K^+ OECTs. (B) Evaluation of repeatability for 3 consecutive calibrations for the same K^+ OECT. All the above experiments were performed in artificial serum at pH 3.8.

IS-OECT photograph

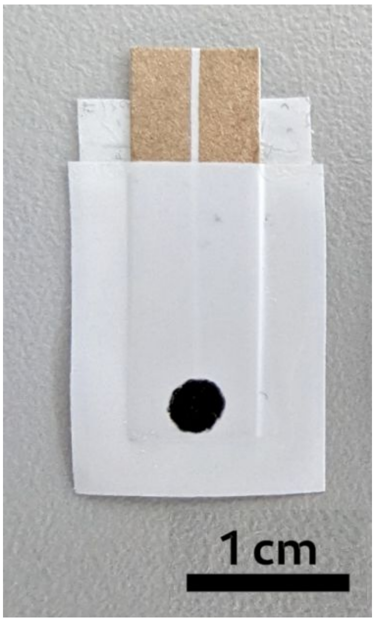

Figure S10. Close-up photography of an IS-OECT. Scale bar correspond to 1 cm.

References

(1) Corba, A.; Sierra, A. F.; Blondeau, P.; Giussani, B.; Riu, J.; Ballester, P.; Andrade, F. J. Potentiometric Detection of Creatinine in the Presence of Nicotine: Molecular Recognition, Sensing and Quantification through Multivariate Regression. *Talanta* **2022**, *246* (April). https://doi.org/10.1016/j.talanta.2022.123473.

(2) Guinovart, T.; Hernández-Alonso, D.; Adriaenssens, L.; Blondeau, P.; Rius, F. X.; Ballester, P.; Andrade, F. J. Characterization of a New Ionophore-Based Ion-Selective Electrode for the Potentiometric Determination of Creatinine in Urine. *Biosens Bioelectron* **2017**, *87*, 587–592. https://doi.org/10.1016/j.bios.2016.08.025.

(3) Clua Estivill, M.; Ait Yazza, A.; Blondeau, P.; Andrade, F. High-Performance Ion-Selective Organic Electrochemical Transistors for the Determination of Potassium in Clinical Samples. *SSRN Electronic Journal* **2022**. https://doi.org/10.2139/ssrn.4157202.

(4) Novell, M.; Parrilla, M.; Crespo, G. A.; Rius, F. X.; Andrade, F. J. Paper-Based Ion-Selective Potentiometric Sensors. *Anal Chem* **2012**, *84* (11), 4695–4702. https://doi.org/10.1021/ac202979j.

(5) Ariadna Dasca Beneito, Pascal Blondeau, Jordi Riu, F. J. A. A Paper-Based Organic Electrochemical Transistor Array with a Simplified Configuration for Simultaneous Multi-Ion Detection. *Talanta* **2024**, 100359. https://doi.org/10.1016/j.talanta.2024.126957.

(6) Analog Devices, Inc. *AD8428 datasheet*. Online.
